# Porcine lung tissue slices: a culture model for PRCV infection and innate immune response investigations

**DOI:** 10.1186/s13568-024-01717-0

**Published:** 2024-05-16

**Authors:** Shuxian Li, Yabin Lu, Shanshan Yang, Caiying Wang, Jing Yang, Xin Huang, Guohui Chen, Yongheng Shao, Maolin Li, Haoyuan Yu, Yuguang Fu, Guangliang Liu

**Affiliations:** 1 State Key Laboratory of Veterinary Etiological Biology, College of Veterinary Medicine, Lanzhou University, Lanzhou Veterinary Research Institute, Chinese Academy of Agricultural Sciences, 1 XuJiaPing, YanChangBu, ChengGuan District, 730046 Lanzhou, Gansu China; 2 College of Veterinary Medicine, Xinjiang Agricultural University, Urumqi, China

**Keywords:** Precision-cut lung slices, Porcine respiratory coronavirus, Innate immune response

## Abstract

Respiratory coronaviruses (RCoVs) significantly threaten human health, necessitating the development of an ex vivo respiratory culture system for investigating RCoVs infection. Here, we successfully generated a porcine precision-cut lung slices (PCLSs) culture system, containing all resident lung cell types in their natural arrangement. Next, this culture system was inoculated with a porcine respiratory coronavirus (PRCV), exhibiting clinical features akin to humans who were infected by SARS-CoV-2. The results demonstrated that PRCV efficiently infected and replicated within PCLSs, targeting ciliated cells in the bronchioles, terminal bronchioles, respiratory bronchioles, and pulmonary alveoli. Additionally, through RNA-Seq analysis of the innate immune response in PCLSs following PRCV infection, expression levels of interferons, inflammatory cytokines and IFN stimulated genes were significantly upregulated. This ex vivo model may not only offer new insights into PRCV infection in the porcine respiratory tract but also serve as a valuable tool for studying human respiratory CoVs infection.

**Supplementary Information:**

The online version contains supplementary material available at 10.1186/s13568-024-01717-0.

## Introduction

Respiratory coronaviruses (CoVs) infections lead to a range of respiratory symptoms, seriously threaten global health and cause significant economic impact. The epidemic of respiratory CoVs in humans, such as severe acute respiratory coronavirus (SARS-CoV), Middle East respiratory syndrome coronavirus (MERS-CoV), and SARS-CoV2, has been linked to spillover of zoonotic diseases from animals (Fung and Liu 2019; Hu et al. 2021; Wong and Perlman 2022). Currently, various animal models have been employed to investigate human respiratory CoVs, including hamster, ferret and non-human primate models, but these models do not fully replicate human diseases (Fan et al. 2022). By contrast, the respiratory organ of porcine is the most similar to humans in anatomy, physiology, and immunology and can be infected by several porcine CoVs (Huang et al. 2024; O’Toole et al. 1989; Sanchez et al. 2019). Porcine respiratory coronavirus (PRCV) has been circulating in pig populations for decades, causing similar clinical features to SARS-CoV-2 infections in human (Hu et al. 2021). Therefore, the study of PRCV infections in the porcine respiratory system will facilitate to better understanding the pathogenesis of human respiratory CoVs.

PRCV was first reported in Belgium in 1984 (Pensaert et al. 1986), and identified as a mutant of transmissible gastroenteritis virus (TGEV) with 227 amino acids deletion of the S protein at the N terminal, which may be related to the change of tissue tropism of the virus. Previous studies have shown that PRCV can infect the nasal mucosa, trachea, bronchi, bronchioles and alveolar macrophages (Cox et al. 1990), leading to clinical symptoms such as dyspnea, shortness of breath, anorexia and growth retardation (Jung et al. 2009). Additionally, PRCV causes broncho interstitial pneumonia and necrotizing alveolitis (Atanasovaa et al. 2008).

Respiratory tract cells are mainly composed of ciliated cells, mucus-producing cells and basal stem cells (Baskerville 1976). Previous studies on coronaviruses have primarily utilized non-physiological cell lines (such as Vero-E6 and swine testis) or primary respiratory epithelial cells and airway organoids (Jiang et al. 2022; Keep et al. 2022). However, these culture systems fail to replicate the complicated airway tract, necessitating the development of a more suitable model for understanding coronavirus infections. Precision-cut tissue (PCT) offers a viable ex vivo culture system with reproducible and defined thickness, containing all cell types of the tissue with intact architecture (Parrish et al. 1995). Precision-cut lung slices (PCLSs) were established as an ex vivo culture system of the lung. Tens to hundreds of slices could were generated from a single lung while maintaining high viability for over a week. Compared to other models, PCLSs possess all resident cells in their natural spatial relationships and structural arrangements. PCLSs can be applied in antiviral drug (Geiger et al. 2023), virus infection (Agraval et al. 2023) and parasite infection (Munyonho et al. 2024). While previous reports have indicated that PCLSs can be cultured in DMEM high glucose, RPMI 1640 and DMEM-F12 (Gerhards et al. 2021; Khan et al. 2021; Tigges et al. 2021), the optimal medium for PCLSs culture in vitro has not been identified.

In this study, we successfully generated porcine PCLSs and investigated their susceptibility to PRCV and the cell tropism of the viruses. Furthermore, we clarified the innate immune responses post-PRCV infection using RNA-Seq. The use of PCLSs presents a novel perspective on the investigations of PRCV infection and may serve as an ideal model to better understand human CoVs as well.

## Materials and methods

### Viruses

The PRCV strain (PRCV/USA/Minnesota/2016, GenBank: KY406735.1) was kindly provided by Professor Song Zhenhui of the Department of Veterinary Medicine at Southwest University in Chongqing, People’s Republic of China. The viral titers of PRCV were determined using swine testis cells through a TCID_50_ assay (50% tissue culture infective dose).

### Establishment of PCLSs culture system

PCLSs were generated as previously reported (Fu et al. 2018). Briefly, lungs from 3-month-old healthy pigs were harvested post-euthanasia, and the upper, middle, and caudal and accessory lobes were isolated. All lobes were infused with 1.5% low-melting-temperature agarose (Agarose, low-melting point, Promega, USA) through the lobar bronchus to maintain the lung’s delicate honeycomb structure. After agarose solidification on ice, cylinder tissues containing a bronchial airway were drilled out using an 8-mm hollow rotating tissue coring tool and sliced to 250 μm thickness using a Krumdieck slicer (Alabama Research & Development, USA). All the generated PCLSs were collected in dishes and incubated at 37 °C and 5% CO_2_ for 1 h to get out the agarose. Next, the highly quality tissue sections were selected and transferred to 24-well plates. PCLSs with the same thickness, round shape, ciliary-beating, smooth edges were identified as highly quality tissue sections. Next, PCLSs were cultured in RPMI1640 (Merck, Germany), DMEM-F12 (Merck, Germany) and DMEM high glucose media (Merck, Germany). Finally, the adenosine triphosphate (ATP) levels of the PCLSs were measured to determine the optimal culture medium.

### Viability analysis of PCLSs

The prepared PCLSs were cultured with different culture media for 72 h. Slices were then collected at the indicated intervals and subjected to the ATP Bioluminescence Assay Kit CLS II kit (Roche Diagnostics, Germany) to measure ATP content according to the manufacturer’s guide. In addition, the slices cultured with RPMI 1640 medium for 72 h were collected and stained using the calcein acetoxymethyl/ethidium homodimer-1 (calceinAM/EthD-1) staining kit (ThermoFisher, USA) to assess PCLS viability, following the manufacturer’s protocol.

### Histological analysis

To determine the intact structure of PCLSs, an H&E (Hematoxylin and eosin) assay was employed and performed as previously reported (He et al. 2022). Briefly, PCLSs were maintained in RPMI 1640 medium without fetal bovine serum (FBS) for 72 h, followed by fixed with tissue fixative solution (4% paraformaldehyde). PCLSs were dehydrated with a gradient of alcohol, and then treated with xylene, followed by embedded in paraffin. Then 4 μm-sections were generated using a Leica RM2245 semi-automatic microtome (Leica Biosystems, Germany). Following, sections were stained with hematoxylin and eosin. Finally, sections were observed under an optical microscope (Lecia, Germany) to analyze the integrity of the slices.

### PRCV infection in PCLSs

PCLSs exhibiting 100% ciliary activity were selected for viral infection. After aspirating the culture medium, the PCLSs were inoculated with PRCV (300 μL RPMI1640/well at 10^5^ TCID_50_/mL) for 2 h at 37 °C and 5% CO_2_. Subsequently, the slices were washed using PBS to remove unattached virions, the culture supernatants and slices were collected at 24 h, 48 h, 72 h and 96 h post-infection to determine viral loads using qPCR and TCID_50_ assays.

### RNA extraction and quantitative real-time PCR

Total RNA of supernatant and PCLSs were extracted using RNAiso Plus (TaKaRa, Japan) according to the manufacturer’s guide. Next, the extracted RNA was reverse transcribed into cDNA for detection of target genes using HiScript III RT SuperMix for qPCR (+gDNA wiper) (Vazyme, China). The copies of PRCV in the infected PCLSs were determined using RT-qPCR assay established in our group (Huang et al. 2019). To validate the RNA-seq results with the RT-qPCR analysis, specific genes expression levels using 2× SYBR-Green Q-PCR Mix (Vazyme, China) and then conducted with the manufacturer’s protocol and normalized to the β-actin expression. The primers used in this study are listed in Table 1.

**Table 1:** Primers for PCR

Name	Primer or probe	Sequence ($5^{\prime}$–$3^{\prime}$)
RIG-1	Forward	GGCTGAAGCCACAGAATA
	Reverse	TCAGTGGTCCGTAATTCC
TLR5	Forward	CCAACACCCTTTCTCCAGCAT
	Reverse	GATAGGACGCACGCCTCTTT
CXCL9	Forward	GACTCAGTGGAACACCTACAGA
	Reverse	TGCAGGAACAACGTCCATTC
IFNAR1	Forward	ACGGGAATCAGAGTCGTCAG
	Reverse	TACCCAGGCGGACAATTTAG
IFN-β	Forward	CTGCTGCCTGGAATGAGAGCC
	Reverse	TGACACAGGCTTCCAGGTCCC
IFN-γ	Forward	TGGTAGCTCTGGGAAACTGAATG
	Reverse	GGCTTTGCGCTGGATCTG
MX2	Forward	ATGCCTAAACCCCGCATGTCGTGGCCTTA
	Reverse	TTACCCCTGTAATGACTGAGCGAATTTGCT
GBP1	Forward	GAAGGGTGACAACCAGAACGAC
	Reverse	AGGTTCCGACTTTGCCCTGATT
GBP2	Forward	AGGGCAGCTCAGCTCAGAAGAA
	Reverse	TGAGTGGCAATGGTTTGGCATC
OAS2	Forward	TCCGCCATTCGGCTACAAAG
	Reverse	CCTGGGAGCCTTCCATTTTG
CXCL9	Forward	GACTCAGTGGAACACCTACAGA
	Reverse	TGCAGGAACAACGTCCATTC
CXCL10	Forward	GTCCAGGTGGCTTATGGAGTC
	Reverse	GTGGGCAAGATTGACTTGCAG
CXCL11	Forward	TGTTCAAAGCGGGAAGGTGT
	Reverse	TGGGATTTAGGCATCTTCGTCC
β-actin	Forward	GCAAATGCTTCTAGGCGGAC
	Reverse	GCGTCCATCACAGCTTCTCA
TGEV-N	Forward	TGCCATGAACAAACCAAC
	Reverse	GGCACTTTACCATCGAAT
	Probe	HEX-TAGCACCACGACTACCAAGC-BHQ1*a*

### Cryosection

The PCLSs were embedded in optimal cutting temperature compound (SAKURA, Japan) in a suitable tissue cryomold and then quickly frozen in liquid nitrogen. The embedded PCLSs are stored at − 80 °C until use. Cryosections of 8-μm thickness were prepared using a Leica CM1950 Cryostat (Leica, Germany), and dried at room temperature (RT) overnight, and stored at − 20 °C until assay.

### Indirect immunofluorescence assay (IFA)

The cryosections were fixed with tissue fixative solution (4% paraformaldehyde) for 30 min at RT and then treated with 100 mM glycine for 10 min. Following washes with PBS, the cryosections were permeabilized with 0.2% TritonX-100 for 20 min. Subsequently, different primary antibodies were diluted with 1% BSA (bovine serum albumin) and then treated with the cryosections for 16 h at 4 °C. For viral particle detection, a mouse anti-TGEV-N protein monoclonal antibody at a 1:500 dilution with 1%BSA was used and then incubated with an anti-mouse IgG (Alexa Fluor 488 conjugate) (CST, USA). To visualize ciliated cells, basal cells and mucus-producing cells, a rabbit anti-Cy3 labeled monoclonal antibody against β-tubulin at a 1:500 dilution with 1%BSA (Sigma-Aldrich, USA), a rabbit anti-cytokeratin 5 (CK5) monoclonal antibody (Abcam, UK) at a 1:500 dilution and a rabbit anti-mucin-5AC antibody at a 1:250 dilution was used, respectively. After PBS washed three times, the secondary antibodies, goat anti-rabbit IgG (Alexa Fluor 594 conjugate) (CST, USA) were diluted at 1:500 with 1%BSA and incubated with the cryosections for 1 h at RT. Finally, nuclei were stained using DAPI (Solarbio, China) for 3–5 min at RT. All sections were embedded in Mounting Medium and subjected to a TCS SP8 confocal microscope for analysis (Leica, USA).

### RNA-seq analysis

PCLSs were infected with or without PRCV and collected at 24 h post-infection. The PCLSs were rapidly frozen in liquid nitrogen and preserved at − 80 °C. The prepared PCLSs were sent to Suzhou GENEWIZ for RNA-seq analysis. The RNA sequencing raw data were processed and mapped based on the *Sus Scrofa* reference genome. The differentially expressed genes (DEGs) between the PRCV-infected group and the mock group were screened using the expression of DEGs more than two times and FDR (qvalue) ≤ 0.05. If the log2Foldchange of a gene was ≥ 1, the differential gene was considered to be up-regulated; otherwise, if the log2Foldchange was ≤ − 1, the differential gene was considered to be down-regulated.

### Statistical analysis

All statistical analyses were conducted using the Student’s *t*-tests, diagram of statistics was made by GraphPad Prism (Version 9.5). All results in the figures are presented as the means±standard errors of the mean (SEMs) from three independent experiments. The significance level (*P* value) is indicated as follows: *, *P* < 0.005; **, *P* < 0.01; ***, *P* < 0.001; ****, *P* < 0.0001.

## Results

### Establishment of ex vivo PCLSs culture model

**Fig. 1:**
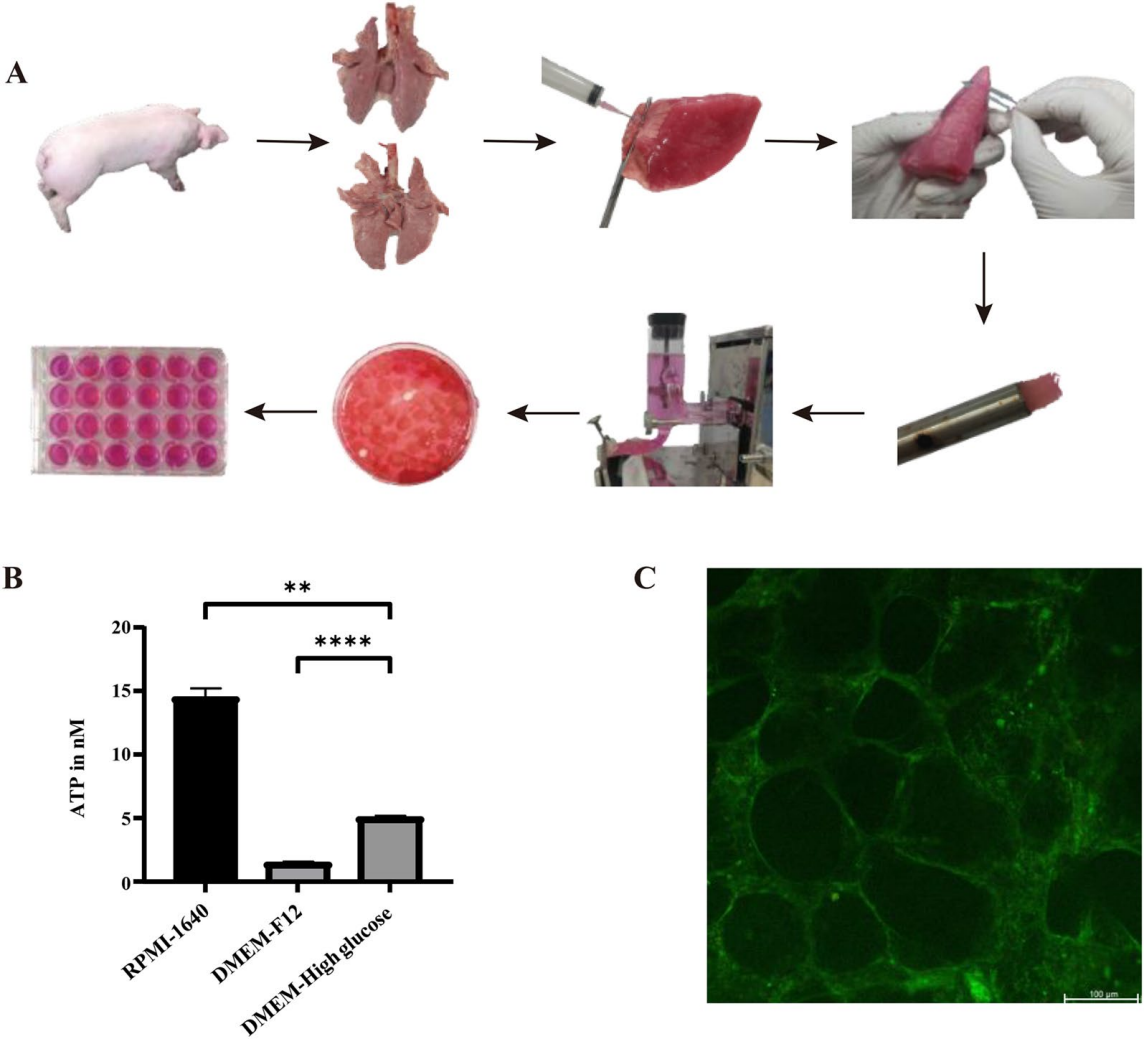
Establishment of ex vivo precision-cut lung slices culture model. The scheme of PCLSs generation. Lobes of the lung were removed from a clinically healthy pig and filled with 1.5% low-melting agarose. Tissues containing a bronchial airway were then stamped out and sliced using a Krumdieck slicer to generate slices. The resulting slices were transformed and cultured in a 24-well plate (**A**). To determine the optimal culture medium, the ATP levels of PCLSs cultured in RPMI-1640, DMEM-F12 or DMEM-High glucose were measured at the indicated time (**B**). A live/dead cell microscopy-based analysis was performed to further analyze the viability of PCLSs cultured with RPMI-1640 for 72 h (**C**)

The scheme for preparing PCLSs is shown in Fig. 1A. To determine the most suitable culture medium, PCLSs were cultured in three different media for specified durations, and their ATP content was measured. The results showed RPMI1640 demonstrating superior performance compared to DMEM-F12 and DMEM-High glucose (Fig. 1B). To further evaluate the viability of PCLSs, a live/dead cell microscopy-based analysis was used, live cells were stained with green, and dead cells were stained with red. The majority of cells stained with green in the PCLSs for 3 days indicated sustained viability of the majority of cells (Fig. 1C). In addition, ciliary activity, as an indicator of viability, was observed to be approximately 100% in PCLSs cultured with RPMI 1640 for 3 days (Supplementary Video 1).

**Fig. 2:**
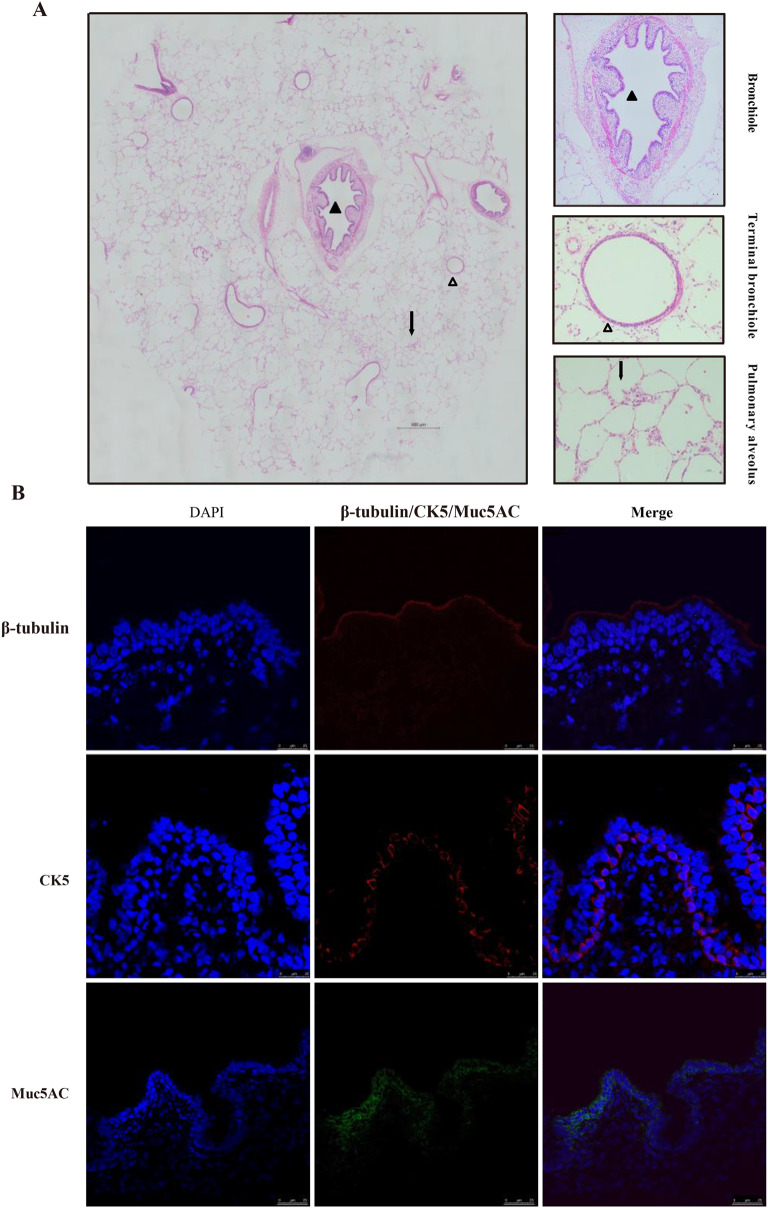
Morphology and main cell types of PCLSs cultured RPMI-1640 for 72 h. To determine the intact structure of PLCSs cultured in RPMI-1640 for 72 h, an H&E assay was employed (**A**). To analyze cell types, cryosections were prepared and labeled with DAPI (nuclear staining, blue), anti-β-tubulin antibody (red), anti-CK5 antibody (red), and anti-MUC5AC antibody (green); ciliated cells (β-tubulin antibody), basal cells (CK5), Mucus-producing cells (MUC5AC) Images were acquired using a TCS SP8-Leica confocal laser scanning microscope (**B**). The scale bar is 25 μm

H&E staining was used to analyze the morphology of PCLSs, revealing the presence of bronchioles, terminal bronchioles, respiratory bronchioles, alveolar ducts, alveolar sacs and pulmonary alveoli in the PCLSs. The epithelium of bronchioles and terminal bronchioles were intact, and there was no necrosis and shedding of epithelium. (Fig. 2A). These results indicate the preservation of the lung’s intact architecture after 72 h of culture. Furthermore, the presence of ciliated cells, goblet cells and basal cells in PCLSs was confirmed using anti-β-tubulin, mucin5AC and CK5 antibodies, respectively (Fig. 2B). These results indicated that the porcine PCLSs culture model was successfully developed in this study and cultured PCLSs remained the same morphology and physiological conditions in vivo.

### PRCV infection in PCLSs

**Fig. 3:**
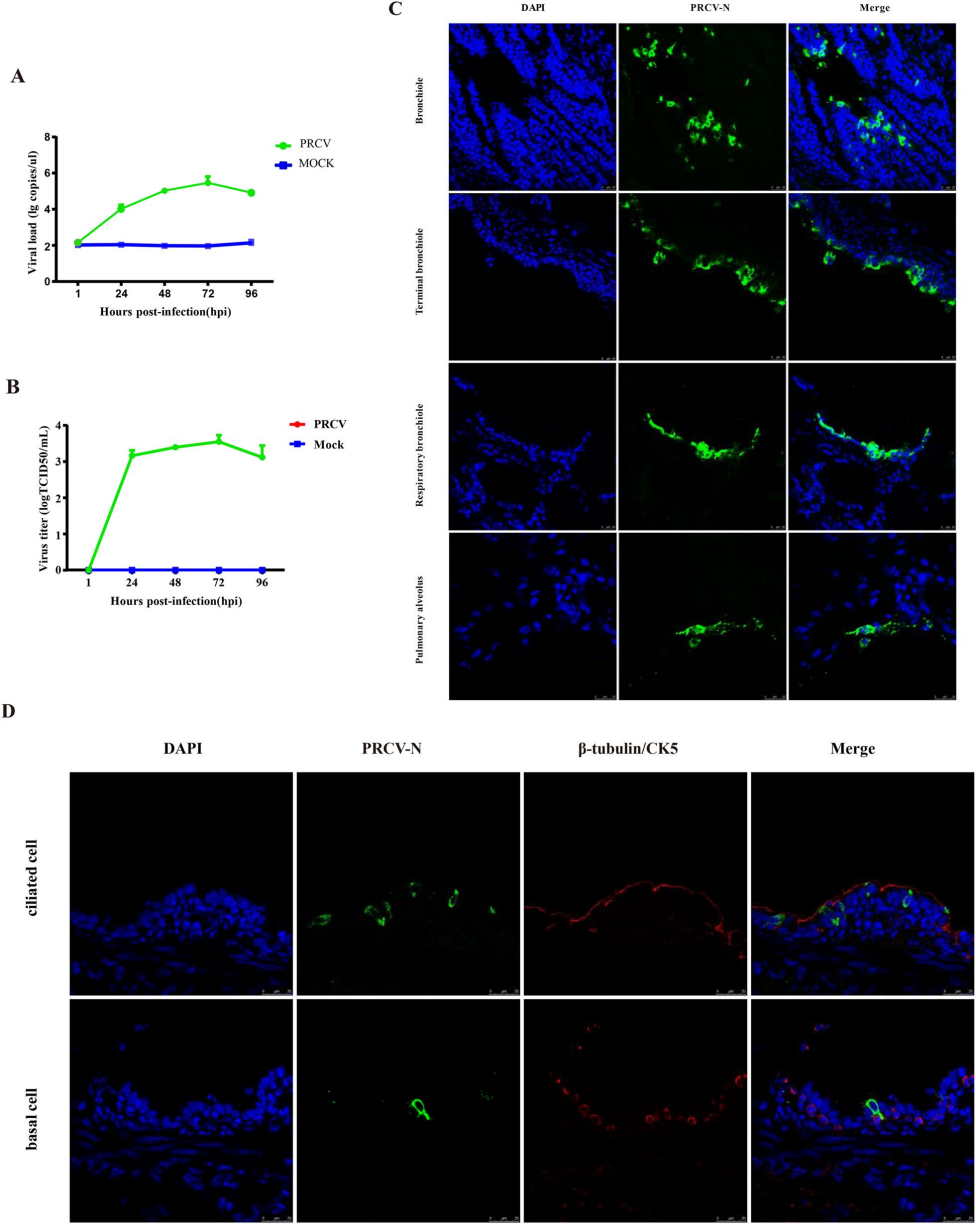
PRCV infection in PCLSs. To analyze the replication of PRCV in PCLSs, viral load in the PCLSs was detected by real-time qPCR (**A**). Supernatants were collected at indicated times post-PRCV infection and determined by TCID_50_ (**B**). To determine whether PRCV (green) infects bronchioles, terminal bronchioles, respiratory bronchioles, and pulmonary alveoli, cryosections were prepared at 48 h post-infection and an IFA assay was employed (**C**). To analyze the cell tropism of PRCV, cryosections were prepared at 48 h post- infection and subjected to IFA staining with antibodies against ciliated cells (red), basal cells (red), and TGEV-N (green) (**D**)

To analyze PRCV replication in PCLSs, culture supernatants and slice samples were collected at the different time points post-infection and analyzed with real-time qPCR and TCID_50_ assay. The viral load peaked at 72 h post-infection (Fig. 3A) and with 3-log-unit increase in viral titer from 1 to 72 hpi (Fig. 3B). The results showed that PRCV could efficiently replicate in PCLSs. The IFA results illustrated that PRCV could infect and replicate in the epithelium of bronchioles, terminal bronchioles, respiratory bronchioles and pulmonary alveoli (Fig. 3C). To identify the cell tropism of PRCV in the lung, the co-staining of the virus and different cell types in the respiratory tract were performed. The results showed that the PRCV mainly infects ciliated cells but not basal cells (Fig. 3D).

### The host defense immunes of PCLS are activated by PRCV infection

**Fig. 4:**
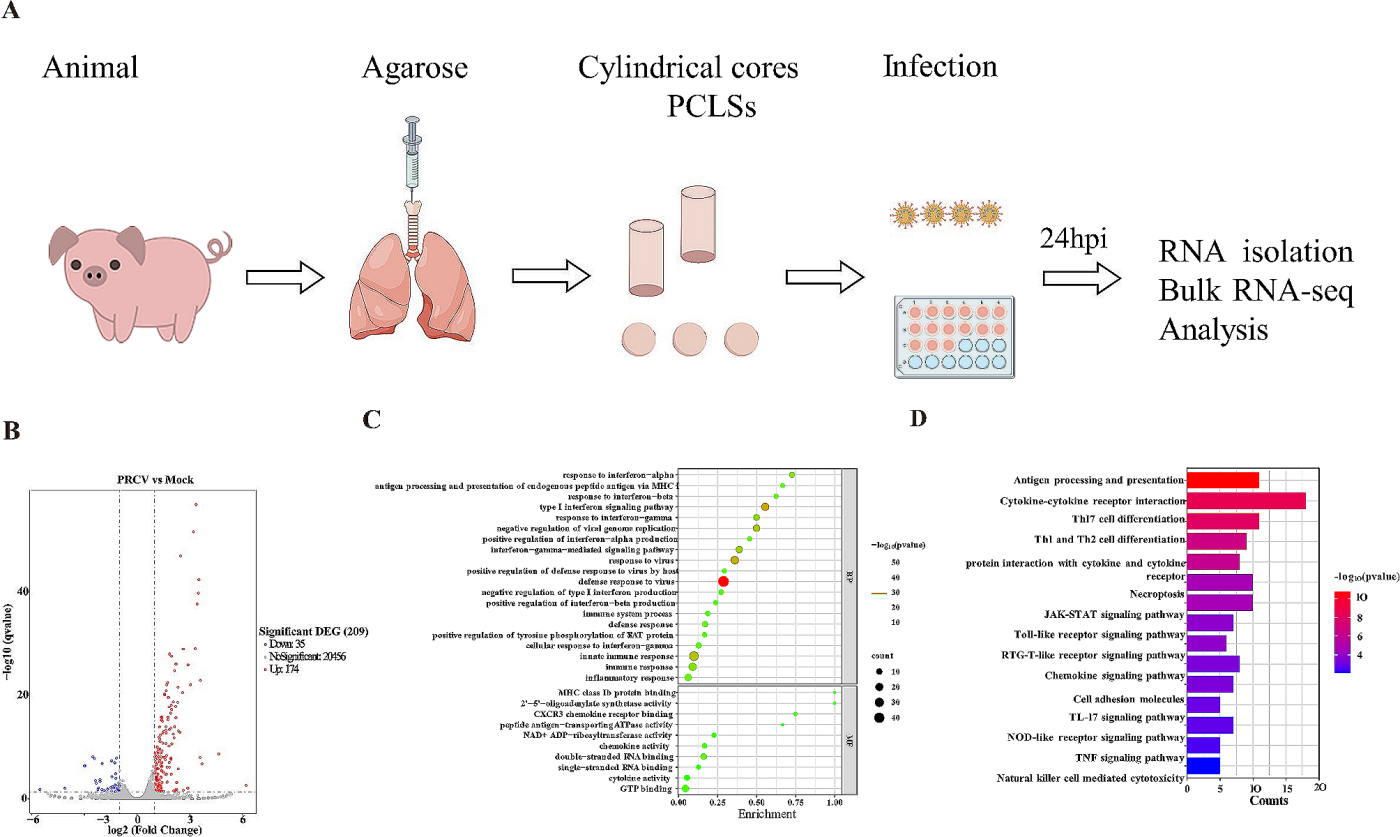
Global differential gene expression analysis of PRCV infection in PCLSs. Scheme of bulk RNA-seq in PCLSs post PRCV-infection. The lung was removed from a healthy pig after anesthesia to generate PCLSs, which were infected with PRCV and subjected to bulk RNA-seq analysis (**A**). The volcano plot shows changes in gene expression induced by PRCV infection in PCLSs (**B**). Terms related to innate immune response were analyzed by GO enrichment analysis of DEGs induced by PRCV infection in PCLSs (**C**). Pathways related to innate immune response were analyzed by KEGG enrichment analysis of DEGs induced by PRCV infection in PCLSs (**D**). *GO* gene ontology, *KEGG* Kyoto encyclopedia of genes and genomes

The innate immune response is essential in combating viral infections. The gene transcriptional profiles in PCLS were examined by RNA-seq after PRCV infection following the workflow shown in Fig. 4A. The results demonstrated that PRCV infection upregulated 174 genes and downregulated of 35 genes in the PCLSs compared to the mock-infected group (Fig. 4B). Gene ontology (GO) and KEGG pathway enrichment analyses were conducted to elucidate the functional implications of DEGs in PCLSs post PRCV infection. Following PRCV infection, the dominant biological processes of the DEGs were associated with the host defense to viruses, such as the interferon signaling pathway, immune response and inflammatory response (Fig. 4C). Furthermore, we utilized the KEGG database to identify the potential cellular pathways involved in PRCV infection. Our analysis revealed enrichment of the KEGG pathways mainly associated with immune processes and host defenses, including cytokine-cytokine receptor interaction, antigen processing and presentation, viral protein interaction with cytokines and cytokine receptors, and the JAK-STAT signaling pathway and so no (Fig. 4D).

**Fig. 5:**
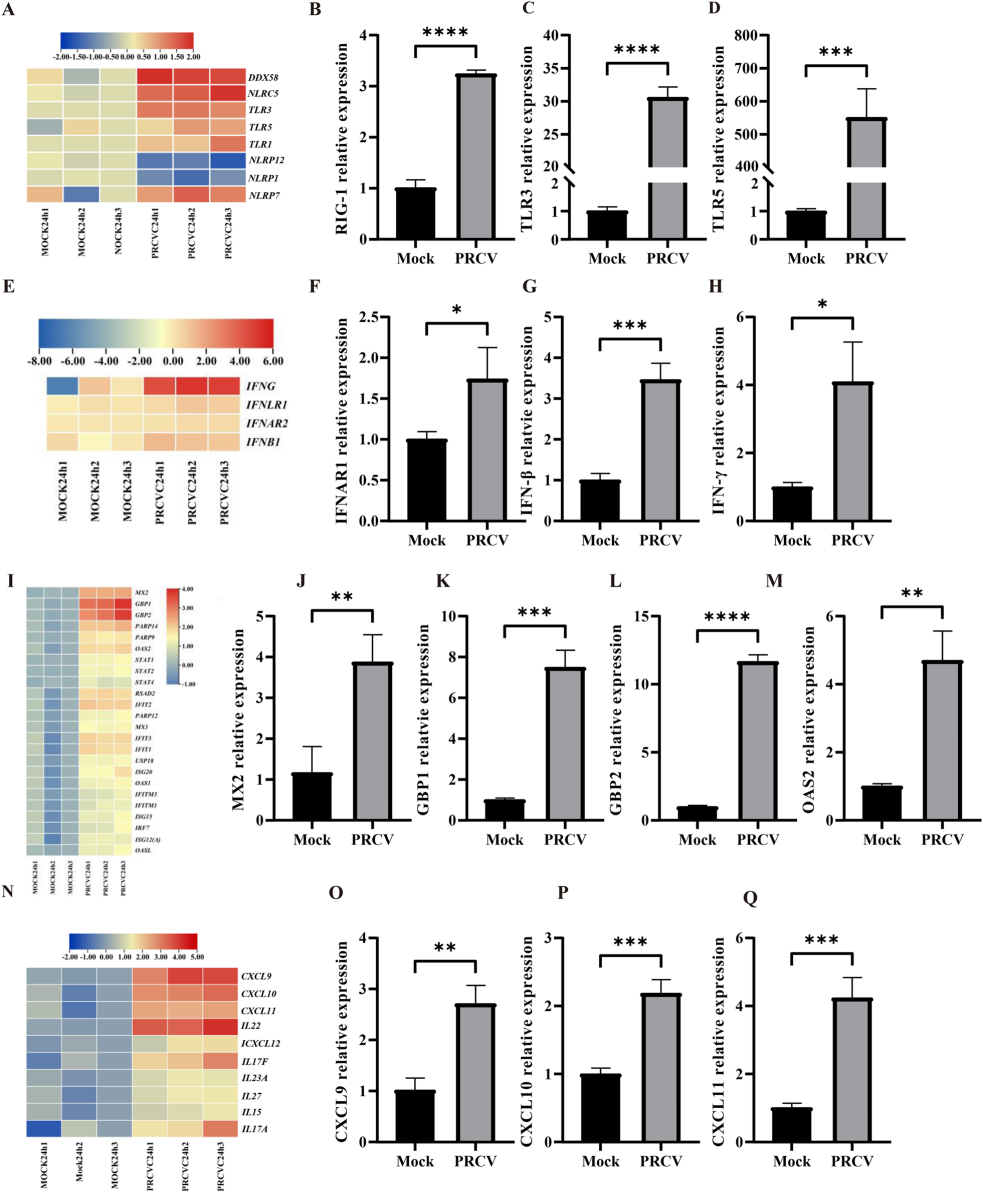
The expression changes of PRRs, IFNs, ISGs and proinflammatory cytokines in PCLSs following PRCV infection. Enriched analysis of PRRs in PCLSs post PRCV infection (**A**). Verification of related PRR expression (RIG-1, TLR3, TLR5) in PCLSs by RT-qPCR (**B**–**D**). Heatmap of IFNs and IFN receptors in PCLSs (**E**). Verification of the expression of IFNAR1 (**F**), IFN-β (**G**), and IFN-γ (**H**) by RT-qPCR. Heatmap of the top 20 upregulated ISGs in PRCV-infected PCLSs (**I**). Identification of related ISG expression by bulk RNA-seq (**J**–**M**). Heatmap of pro-inflammatory cytokines in PCLSs (**N**). Verification of the expression of proinflammatory cytokines (CXCL9, CXCL10, CXCL11) in PCLSs by RT-qPCR (**O**–**Q**). Results of real-time PCR are expressed as the mean ± SEM from three independent experiments. Results of real-time PCR were analyzed by the *t*-test. *, *P* < 0.05; **, P < 0.01; ***, *P* < 0.001; ****, *P* < 0.0001

Upon viral invasion, host pattern recognition receptors (PRRs) detect viral genetic material and proteins, leading to the activation of immune responses such as interferons (IFN) and inflammatory cytokine production. The heatmap analysis revealed that the RIG-I (DDX58), TLR3 and TLR5 were upregulated following PRCV infection (Fig. 5A). This upregulation was further confirmed by RT-qPCR, showing the same trends with RNA-seq results (Fig. 5B–D). Subsequently, IFN induction led to an increase in the expression of interferon-stimulated genes (ISGs), playing a crucial role in the antiviral response. Specifically, PRCV infection led to the upregulations of type I, type II and type III IFNs (Fig. 5E), which was proved by RT-qPCR (Fig. 5F–H). Additionally, PRCV-infected PCLSs produced a massive expression of ISGs compared to the mock group (Fig. 5I), as validated by RT-qPCR analysis of GBP1, GBP2, OAS2 and MX2 (Fig. 5J–M). In vivo, PRCV infection resulted in bronchointerstitial pneumonia (Jung et al. 2007; Van Reeth et al. 1999). We next analyzed the transcriptional expression of pro-inflammatory cytokines following PRCV infection in PCLSs. Our analysis revealed a significant upregulation of pro-inflammatory cytokines and chemokines in PRCV-infected PCLSs compared to the mock group (Fig. 5N). The RT-qPCR results for several pro-inflammatory cytokines were randomly consistent with RNA-seq results (Fig. 5O–Q). Collectively, these results suggested that the host defense immune responses were successfully initiated in the porcine PCLSs culture system developed in this study, implying that this culture model is an ideal platform to mimic the viral infection and innate immune responses in vivo.

## Discussion

PRCV is a pathogen capable of infecting the porcine lung and causing respiratory disease complex (Opriessnig et al. 2011). Given its similarities to respiratory coronaviruses infection in the human lung (Keep et al. 2022), PRCV serves as an ideal model for studying coronavirus infections in the respiratory tract. However, there is currently no suitable culture model available to simulate the natural lung environment. PCLSs, an ex vivo model, have been effectively used to study pathogenic lung infections, including those caused by paramyxoviruses, rhinoviruses, adenoviruses and influenza viruses (Viana et al. 2021). In the study, we successfully established a porcine PCLSs ex-vivo model. Due to different cultural medium used in previous PCLSs culturing and different cell viability exhibited, we screened the optimal medium for culturing PCLSs. Our results indicated that RPMI 1640 was the optimal culture medium based on their ATP content and cell viability of PCLSs. Furthermore, we analyzed the viability and structure of slices cultured with RPMI 1640 for 72 h, and observed that the PCLSs retained its intact structure and cellular activity. Meanwhile, we observed that the ciliary activity of the PCLSs was 100% after cultured with the medium for 72 h using an optical microscope. The results of histology, IFA and optical microscope observation demonstrated that PCLSs remained their natural property under the culture condition.

The PCLSs were infected with PRCV for specific durations, the TCID_50_ and RT-qPCR results showed efficient replication in this culture model. Compared with the viral load of PRCV infection in the 3D airway organoid (Jiang et al. 2022), no significant differences in the PRCV infected PCLSs. These indicated the suitability of PCLSs as a culture system for PRCV infection. Previous studies have reported that PRCV can infect various cell types, including bronchiolar epithelial cells, type I pneumocytes, type II pneumocytes and septal macrophages (Cox et al. 1990). In addition, PRCV also can infect non-ciliated cells and non-mucus-producing cells in the porcine respiratory tract (Hanna and Malgorzata 2021). In this study, we also observed that PRCV mainly infects bronchiolar epithelial cells, particularly ciliated cells. Additionally, PRCV was detected in type II pneumocytes but not in type I pneumocytes. The reason for these differences remains unclear, and it is possible that different viral strains may result in changes in cell tropism, necessitating further exploration.

The innate immune response provides the first defense line against viral infections. This study investigated the innate immune response induced by PRCV infection in PCLSs using RNA-Seq. We observed that the response of PRRs was induced by PRCV infection, which is consensus with previous reports (Charley et al. 2006; Van Reeth et al. 1999). Upregulation of PRRs triggers the production of effectors including IFNs, ISGs, pro-inflammatory factors, and chemokines. The results indicated an increase in related molecules of the innate immune response following PRCV infection. Upregulation of proinflammatory cytokines induced by PRCV infection in the PCLSs was consistent with pathological changes (Bronchopneumonia) caused by the PRCV infection in vivo, indicating PCLSs is an ideal ex vivo model of porcine lung. Notably, the PRRs and effectors induced by PRCV in PCLSs differ from those in airway organoids (AOs) (Jiang et al. 2022), potentially due to differences in the structure, cell types, and PRCV strains. Further investigation is required to validate these differences.

The ex vivo culture system PCLSs accurately replicate the natural arrangement of lung epithelial cells and can be cultured long-term with high cyto-activity. They are valuable for analyzing the cell tropism and innate immune response to viral infections. However, PCLSs have limitations such as the inability for serial in vitro passage, unlike immortalized cell lines and the complexity of mechanistic investigations. To sum up, PCLSs are a useful tool to analyze porcine respiratory virus infections and have the potential for investigating human respiratory CoV infections.

In this study, PCLSs were successfully generated and validated the cell tropism, replication and innate immune response of PRCV infection. This culture system offers a promising model to analyze the infection of porcine or human respiratory infections.

### Supplementary Information

Additional file1 (AVI 441196 kb)

## Data Availability

The data supporting the results of this study are available in the paper and supplementary. The raw data of RNA-sequencing have been submitted to NCBI (National Center for Biotechnology Information) and data can be retrieved with accession numbers PRJNA1070877.
